# Complement Factor H Levels Associate With *Plasmodium falciparum* Malaria Susceptibility and Severity

**DOI:** 10.1093/ofid/ofy166

**Published:** 2018-07-20

**Authors:** Anna E van Beek, Isatou Sarr, Simon Correa, Davis Nwakanma, Mieke C Brouwer, Diana Wouters, Fatou Secka, Suzanne T B Anderson, David J Conway, Michael Walther, Michael Levin, Taco W Kuijpers, Aubrey J Cunnington

**Affiliations:** 1 Department of Immunopathology, Sanquin Research and Landsteiner Laboratory of the Academic Medical Centre, University of Amsterdam, Amsterdam, the Netherlands; 2 Department of Pediatric Hematology, Immunology and Infectious Diseases, Emma Children’s Hospital, Academic Medical Centre, Amsterdam, the Netherlands; 3 Medical Research Council Unit, The Gambia at London School of Hygiene and Tropical Medicine; 4 Department of Pathogen and Molecular Biology, London School of Hygiene and Tropical Medicine, London, United Kingdom; 5 Section of Paediatrics, Imperial College London, London, United Kingdom; 6 Department of Blood Cell Research, Sanquin Research and Landsteiner Laboratory of the Academic Medical Centre, University of Amsterdam, Amsterdam, the Netherlands

**Keywords:** complement Factor H, malaria, severity, susceptibility

## Abstract

**Background:**

*Plasmodium falciparum* may evade complement-mediated host defense by hijacking complement Factor H (FH), a negative regulator of the alternative complement pathway. Plasma levels of FH vary between individuals and may therefore influence malaria susceptibility and severity.

**Methods:**

We measured convalescent FH plasma levels in 149 Gambian children who had recovered from uncomplicated or severe *P. falciparum* malaria and in 173 healthy control children. We compared FH plasma levels between children with malaria and healthy controls, and between children with severe (n = 82) and uncomplicated malaria (n = 67). We determined associations between FH plasma levels and laboratory features of severity and used multivariate analyses to examine associations with FH when accounting for other determinants of severity.

**Results:**

FH plasma levels differed significantly between controls, uncomplicated malaria cases, and severe malaria cases (mean [95% confidence interval], 257 [250 to 264], 288 [268 to 309], and 328 [313 to 344] µg/mL, respectively; analysis of variance *P* < .0001). FH plasma levels correlated with severity biomarkers, including lactate, parasitemia, and parasite density, but did not correlate with levels of PfHRP2, which represent the total body parasite load. Associations with severity and lactate remained significant when adjusting for age and parasite load.

**Conclusions:**

Natural variation in FH plasma levels is associated with malaria susceptibility and severity. A prospective study will be needed to strengthen evidence for causation, but our findings suggest that interfering with FH binding by *P. falciparum* might be useful for malaria prevention or treatment.

Malaria accounts for a huge global burden of disease. *Plasmodium falciparum* infections account for most of this burden, and most attributable deaths occur in young children in sub-Saharan Africa. Three clinical syndromes of severe malaria, occurring alone or in combination, predict the majority of deaths in children: cerebral malaria (characterized by coma), severe anemia, and respiratory distress (which usually indicates metabolic acidosis and hyperlactatemia) [1]. Why some children develop severe malaria and others do not is poorly understood.

The complement system is activated as part of the innate immune response to malaria [2]. *P. falciparum* is vulnerable to complement-mediated defenses when parasites exit host cells to invade new erythrocytes or undergo sexual reproduction [2]. Rapid activation of complement opsonizes invading parasites for phagocytosis or lyses them directly and stimulates the adaptive immune response. Although complement activation is likely to be protective, excessive activation would be detrimental to the host, contributing to dysregulated inflammation and destruction of uninfected erythrocytes [2]. To prevent bystander damage, the complement system is tightly regulated by various fluid phase and membrane-bound regulators. One important regulator protecting host endothelial cells and erythrocytes is the alternative pathway inhibitor factor H (FH), which normally circulates in the blood at ~300 µg/mL [3].

FH plays an essential role in regulating complement activity, and its genetic deficiency results in atypical hemolytic uremic syndrome [3]. However, many microbes have evolved to hijack FH to minimize complement activation on their outer membranes. A well-characterized example is *Neisseria meningitidis,* for which high FH plasma levels increase susceptibility to invasive disease [4, 5]. *P. falciparum* can also recruit FH to its surface, protecting parasites from complement and raising the possibility that variation in plasma levels of FH may also influence outcomes in malaria [6–9].

Although in vitro studies on the mechanisms of FH binding to the malaria parasite demonstrate a plausible mechanism of evading host defense, clinical studies are needed to determine the relevance to disease outcomes. As it has previously been shown that complement activation levels return to normal within a month after a malaria episode [10], we determined convalescent FH plasma levels of children who required hospital treatment for *P. falciparum* malaria as a proxy for the steady-state situation preceding infection. We compared levels with healthy community controls and between those who had suffered uncomplicated vs severe malaria.

## METHODS

### Patients and Samples

Subjects were Gambian children (<16 years old) recruited from the Greater Banjul region, where malaria transmission is seasonal and relatively low [11]. Convalescent heparinized plasma was obtained 28 days after presentation from children (median age [interquartile range {IQR}], 5 [3–8] years) who had received hospital treatment for *P. falciparum* malaria with a parasite density >5000/µL [12–14]. Severe malaria was classified by the presence of 1 or more of the following: severe anemia (SA; hemoglobin < 5 g/dL), hyperlactatemia (LA; lactate > 5 mM), cerebral malaria (CM; Blantyre Coma Scale < 3), or prostration (SP) [14]. Samples were collected from healthy community control children of similar age (median age [IQR], 3.9 [2.4–5.5] years), recruited from the same region between 2013 and 2014. Control children were not tested for asymptomatic parasitemia. The study was approved by the Gambia Government/UK Medical Research Council Joint Ethics Committee and performed in accordance with the Declaration of Helsinki.

### Factor H and *P. falciparum* Histidine Rich Protein 2 (PfHRP2) Emzyme-Linked Immunosorbent Assay

Enzyme-linked immunosorbent assays (ELISAs) to measure Factor H and PfHRP2 were performed as previously described [14, 15].

### Statistics

Analysis was performed with GraphPad Prism v7.02 (GraphPad Software, La Jolla, CA, USA). Differences in mean values between groups were assessed by unpaired *t* test or 1-way analysis of variance (ANOVA) with Tukey’s multiple comparisons test. Correlations were assessed using a nonparametric Spearman’s correlation test. Logistic and linear regression models were developed using the GLM function in R. The best model was assessed by the Akaike Information Criterion.

## RESULTS

To determine whether FH levels may associate with malaria susceptibility and severity, we measured FH in convalescent samples from children with uncomplicated (n = 67) and severe (n = 82) malaria and in healthy community controls (n = 173). Because severe malaria is more common in younger children we first tested whether FH levels were associated with age and found that they were not significantly correlated (n = 322, *r*_s_ = –.034, *P* = .54), as was expected based on a previous study in healthy children [16].

**Figure 1.**
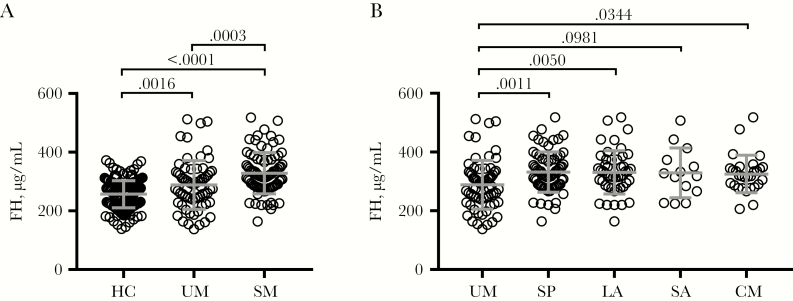
Factor H (FH) plasma levels associate with malaria susceptibility and severity. A, FH was measured by in-house enzyme-linked immunosorbent assay in samples from healthy community control children (HC; n = 173) and in samples obtained 28 days after hospital presentation from children with uncomplicated malaria (UM; n = 67) and severe malaria (SM; n = 82). *P* values indicate Tukey’s multiple comparisons test performed after 1-way analysis of variance. B, Severe malaria was categorized based on major criteria of severity: severe prostration (SP; n = 69), hyperlactatemia (LA; n = 48), severe anemia (SA; n = 14), and cerebral malaria (CM; n = 29). Due to overlapping clinical features, depicted groups are not mutually exclusive. Unpaired *t* tests compare the mean of each group with uncomplicated malaria. Bars indicate mean ± SD.

We then tested whether FH levels were different between healthy controls and malaria cases. FH levels were lowest in healthy controls, intermediate in convalescent uncomplicated malaria cases, and highest in convalescent severe malaria cases (mean [95% confidence interval {CI}], 257 [250 to 264], 288 [268 to 309], and 328 [313 to 344] µg/mL, respectively; ANOVA *P* < .0001) (Figure 1A). This indicates that both the need for hospital treatment for malaria and severity of malaria associate with FH.

Severe malaria can manifest in several overlapping syndromes; therefore, we examined whether the higher levels of FH in those with severe malaria were driven by any particular clinical phenotype. FH levels were similar in all major severe malaria phenotypes and were all significantly higher than in uncomplicated malaria, with the exception of the small group with SA (mean difference [95% CI] compared with uncomplicated malaria: SP, 43 [18 to 69] µg/mL; LA, 43 [13 to 73] µg/mL; SA, 41 [–8 to 90] µg/mL; CM, 37 [2 to 71] µg/mL) (Figure 1B).

**Figure 2.**
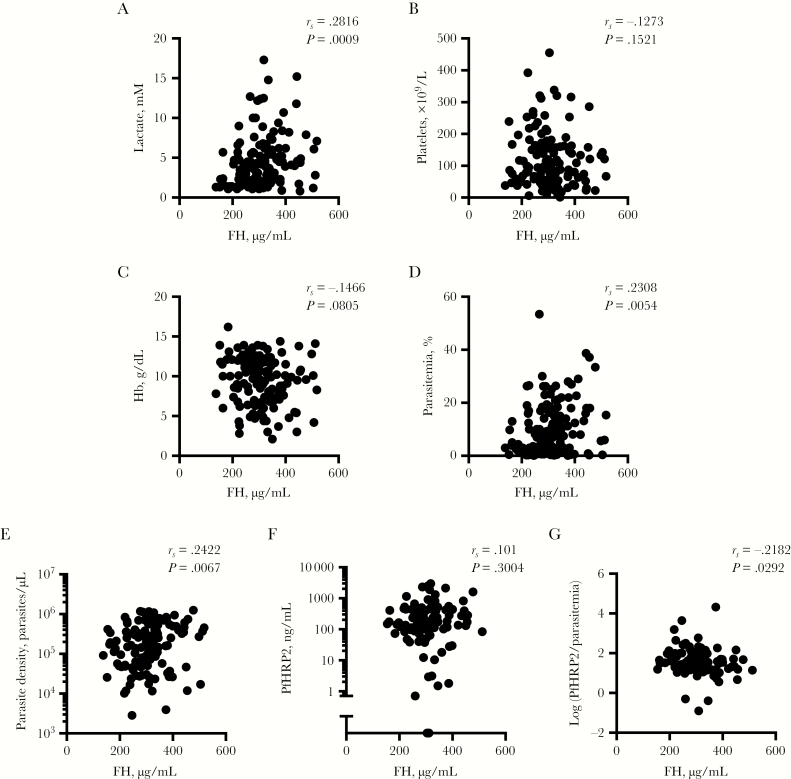
Factor H (FH) plasma levels associate with severity markers. A–D, Correlations of FH plasma levels with severity markers at the time of presentation to hospital: (A) lactate, (B) platelets, (C) hemoglobin (Hb), (D) parasitemia (% of infected erythrocytes in blood film), (E) parasite density, (F) PfHRP2, (G) sequestration index [log (PfHRP2/parasitemia)]. Spearman’s correlations.

To further investigate the association between FH and severity, we assessed relationships between convalescent FH and several host- and parasite-derived markers measured on admission. Lactate concentrations correlated significantly with FH levels, but platelets and hemoglobin did not (Figure 2A–C). Markers of circulating parasite load, parasitemia assessed from blood film and parasites/µL, showed significant positive correlations with FH levels (Figure 2D, E).

Measures of circulating parasite load do not quantify additional cyto-adherent parasites sequestered in the tissue microvasculature. Total body parasite load can be assessed using the plasma concentration of the soluble parasite protein PfHRP2, which reflects both sequestered and circulating parasite numbers and is more predictive of clinical outcome of infection [14, 17]. Surprisingly, there was no correlation between PfHRP2 concentration and FH (Figure 2F). As an index of sequestration, we calculated the ratio of PfHRP2 to parasitemia (higher values would be expected to indicate relatively more sequestration) and found that this was negatively correlated with FH (Figure 2G).

In logistic regression models, age, PfHRP2, and FH all predicted severity, and a multivariate model incorporating all 3 indicated that that they have independent associations with outcome (Table 1). Linear regression models indicated that age, PfHRP2, and FH were also independently associated with blood lactate concentration (Table 2).

**Table 1.** Logistic Regression Models to Predict Severity

Model	Variable	No.	Log Odds	SE	*P*	AIC
Age	Age	149	–0.37	0.069	5.3 × 10^-8^	165
Log PfHRP2	Log PfHRP2	104	1.06	0.25	2.2 × 10^-5^	127
FH	FH	149	0.0066	0.0023	.0047	205
Age + log PfHRP2 + FH	Age	104	–0.45	0.11	2.68 × 10^-5^	87
	Log PfHRP2		0.91	0.27	.00098	
	FH		0.01	0.0045	.00133	

Age, PfHRP2, and FH were assessed individually or combined in a multivariate model to predict severity.

Abbreviations: AIC, Akaike Information Criterion; FH, Factor H.

**Table 2.** Linear Regression Model to Predict Blood Lactate Concentration

Model	Variable	No.	Coefficient	SE	*P*	AIC
Age	Age	138	–0.11	0.015	3.02 × 10^-11^	263
Log PfHRP2	Log PfHRP2	100	0.23	0.036	6.7 × 10^-9^	166
FH	FH	138	0.002	0.00077	.0094	301
Age + log PfHRP2 + FH	Age	100	–0.073	0.012	7.4 × 10^-8^	131
	Log PfHRP2		0.18	0.030	3.5 × 10^-8^	
	FH		0.0021	0.00063	.0011	

Age, PfHRP2, and FH were assessed individually or combined in a multivariate model to predict ln (lactate).

Abbreviations: AIC, Akaike Information Criterion; FH, Factor H.

## DISCUSSION

Despite the huge evolutionary pressure malaria has exerted, surprisingly few host factors have been convincingly shown to influence susceptibility or severity. Of the host factors with the strongest evidence, almost all involve the erythrocyte [18]. Our results suggest that natural variation in FH levels is another determinant of malaria susceptibility and severity.

All children in malaria-endemic countries are potentially susceptible to *P. falciparum* infection, but not all of these infections will result in symptomatic malaria. The likelihood of an infection causing symptomatic disease is dependent on parasite density [19]. As we only included children seeking hospital treatment with >5000 parasites/µL in our study, we ensured high specificity of the malaria diagnosis.

By comparing healthy children with those who had suffered a definite malaria episode, we expected that even relatively small differences in FH between the groups would indicate an association between FH and susceptibility to symptomatic malaria: the outcome of a new infection in the controls could be either asymptomatic or symptomatic, whereas our malaria cases are all symptomatic. Thus our results indicate that higher levels of FH are associated with susceptibility to symptomatic malaria. Among subjects with malaria, the associations of FH with clinical features of severe disease, lactate concentration and circulating parasite load, are consistent with the hypothesis that higher levels of FH also predispose to severe malaria. Indeed, multivariate models suggested that the association of FH with severity was independent of both age and parasite load.

Although high FH levels may allow parasites to evade complement-mediated clearance, high levels may also limit endothelial activation and expression of adhesion molecules involved in sequestration of parasitized erythrocytes [2]. If high FH levels favor parasite multiplication but restrict sequestration, one might expect a stronger correlation with circulating parasite load than with PfHRP2 concentration (which reflects both circulating and sequestered parasites). This would also be consistent with the negative correlation observed between FH and the sequestration index (PfHRP2:parasitemia).

Although total body parasite load is a strong predictor of severity, it is not the only determinant [20]. The rapidity of parasite growth in the circulation may also contribute to severity if the consequent pro-inflammatory responses occur faster than regulatory and protective responses, which are necessary to limit immunopathology. Our study design precluded analysis of nonsurvivors, so we cannot exclude the possibility that high FH levels modulated the risk of death.

The main limitation of our study is that we used convalescent plasma samples, which we believe are representative of the pre-infection status of our subjects. We cannot exclude that differences in FH levels could represent a residual effect of infection, although we think this is unlikely because of the lack of association with PfHRP2 concentrations. A large prospective study would be required to confirm that natural variation in FH levels definitely does predict susceptibility, severity, and mortality from malaria. Further studies will be needed to clarify the relative roles of complement activation and regulation in malaria, but interference with parasite FH binding might be considered as an adjunctive approach to limit parasite growth, enhance parasite clearance, and tackle drug resistance.
